# A scoping review of the psychosocial aspects of infertility in African countries

**DOI:** 10.1186/s12978-024-01858-2

**Published:** 2024-08-23

**Authors:** R. Roomaney, M. Salie, D. Jenkins, C. Eder, M. J. Mutumba-Nakalembe, C. Volks, N. Holland, K. Silingile

**Affiliations:** 1 Department of Psychology, Stellenbosch University, Private Bag X1, Matieland, Stellenbosch, 7602 South Africa; 2 University of Waterloo, Waterloo, ON Canada; 3 La Trobe University, Melbourne, Australia; 4 Department of Psychology, University of the Western Cape, Bellville, South Africa

**Keywords:** Infertility, Scoping review, Africa, Psychosocial, Review

## Abstract

Infertility refers to the inability to conceive after 12 months of regular, unprotected sexual intercourse. Psychosocial aspects of infertility research are predominant in developed countries. A scoping review of psychosocial aspects of infertility research conducted in Africa between 2000 and 2022 was conducted. Twelve databases and grey literature were searched for articles. Studies were included if they were published in English and included findings from patients diagnosed with primary or secondary infertility. A total of 2 372 articles were initially found and screening resulted in 116 articles being included in the scoping review. Most of the studies (81%) were conducted in Nigeria, Ghana and South Africa. Psychosocial aspects explored included quality of life, barriers to treatment, attitudes and stigma, and sociocultural and religious aspects of infertility, among others. The review maps published psychosocial research in the context of infertility in Africa and identifies gaps for future research.

## Plain language summary

Our aim was to review published studies on psychological and social research conducted among men and women who were seeking treatment for infertility in Africa between 2000 and 2022.

We initially found 2 373 articles that seemed appropriate but after screening these articles only included 116 in this review.

We found that in Africa, Nigeria, Ghana and South Africa produced the most studies on the psychological and social impact of infertility.

Common areas of research include exploring patients quality of life, barriers to seeking fertility treatment, stigma and attitudes around infertility, social, cultural and religious issues relating to infertility.

This review is therefore helpful in understanding where psychological and social research on infertility is being conducted, what it is focused on and what the gaps in research are.

## Background

Infertility is defined as a disease of the reproductive system and is defined as “a failure to achieve pregnancy after 12 months or more of regular, unprotected sexual intercourse” [1]. While infertility is a global issue, affecting approximately 8–12% of the global population, the majority of infertile couples reside in developing countries [2].

Infertility affects the personal and social worlds of couples. Infertility and its associated treatment yield numerous psychosocial challenges, as documented in systematic reviews by [3–7]. The experience of infertility is emotionally taxing on a couple as parenting is a major life transition and an important step for couples [8–10]. In addition, the inability to conceive naturally places undue strain on the relationship [9–11], is a lengthy and costly process [12], and results in financial burden and further strain [13]. Although the emphasis is placed on couples’ experiences, research has indicated that infertility impacts the individual within the couple as well. Both partners have their own experiences of loss and flawed identities [8]. Male partners tend to experience heightened distress when infertility is due to the male factor [8], and women experience infertility as a greater life crisis than men [14]. Furthermore, there is a significant association between anxiety and depression among infertile women compared to fertile women [7, 11, 15]. Similarly, men diagnosed with infertility experience higher levels of distress than fertile men [7]. These psychological effects of infertility on individuals have prompted discussions on a possible bidirectional relationship between psychological factors and infertility [13, 16].

There are important social aspects to infertility as well. Social labelling of infertility is rife in societies [13, 15], especially in African countries, where fertility has a high social value. Within many of these contexts, infertile couples often face social exclusion, and these marriages impacted by infertility frequently end in divorce [15, 17]. In particular, women in African countries often bear the brunt of infertility as they are blamed when they fail to become pregnant [18]. Infertility then results in male partners divorcing their wives, taking on a second wife or engaging in extramarital affairs; all actions are considered socially acceptable even when the cause of infertility has not been identified [18].

The psychosocial impact of infertility is well-known [16]. While several studies have been conducted in Africa, this research needs to be more cohesive. The scoping review aims to provide an overview of findings related to psychosocial aspects of infertility among men and women in Africa.

## Method

The protocol for this study was published in BMJ open on 28 May 2021 (https://pubmed.ncbi.nlm.nih.gov/34049906/). The protocol describes the method for a larger study where psychosocial aspects of infertility in developing countries are reviewed. After conducting the review, we concluded that the findings were too dense to be reported in one paper and had therefore split the findings into three papers, representing three regions, namely (1) Africa; (2) Middle East and Asia; and (3) Latin America and the Caribbean. This allows us to provide a thorough review of each developing region.

### Search strategy

The authors developed a search strategy in consultation with a specialist librarian. Literature searches were conducted on 12 databases: Academic Search Premier, African digital repository (Sabinet), CINAHL, Clinical Key, Cochrane library, Google Scholar, PsycArticles, PsycInfo, Pubmed, Scopus, Web of Science, and Proquest. The following search strings were used: Concept 1: terms related to infertility—Infertility OR Involuntary childlessness OR Assisted reproduction OR ART OR Medically Assisted Reproduction OR MAR OR Secondary infertility. AND concept 2: terms related to psychosocial aspects—culture OR religion OR spiritual* OR religious OR stigma OR psychosocial needs OR counselling OR family OR psychosocial impact OR maternal needs OR paternal needs OR tradition OR depress* OR anxiety OR Psychosocial Support Systems [mesh]). AND concept 3: developing countries OR (name of each developing country in Africa).

In addition, grey literature (e.g., unpublished theses and dissertations) were searched, and articles from other sources (such as reference lists) were added. Finally, we emailed researchers in the field and asked them to submit any peer-reviewed, published research. The searches were conducted between August and September 2022.

The following inclusion criteria were used: studies reported in English between 2000 and 2022; both primary and secondary studies; participants included both males and females diagnosed with primary or secondary infertility; qualitative and quantitative studies; data must have been collected in African countries. In addition, studies in languages other than English, theoretical papers and conference proceedings were excluded from the review.

### Study selection

Search results were exported to Rayyan (http://rayyan.qcri.org), where they were further evaluated. A total of 2372 articles were imported to Rayyan, and 1114 duplicates were detected and removed. The remaining abstracts (n = 1258) were each screened by two reviewers (RR and MS) to determine their suitability for inclusion in the review. Only articles deemed suitable for review by both reviewers were included in the next phase. The review resulted in 96 articles being included and 1110 being excluded. Finally, a third reviewer (DJ) assessed articles conflicted by both reviewers (n = 52) to determine suitability for inclusion. This process was a blind review, and 116 articles were deemed suitable for this scoping review.

### Charting the data and reporting the findings

All authors participated in this phase of the process. Reviewers read the full-text versions of the 116 articles and charted the data. The data were charted using a charting form. The comprehensiveness of the form was evaluated by all reviewers who independently charted the same five studies using this form. The team then met and compared the consistency of data extraction using the form. The charting form was deemed appropriate, and no changes were made to the chart. Data extraction appeared consistent, and all the authors then charted the remaining 111 articles. All authors then summarised the data, as reported in the next section.

## Results

**Fig. 1:**
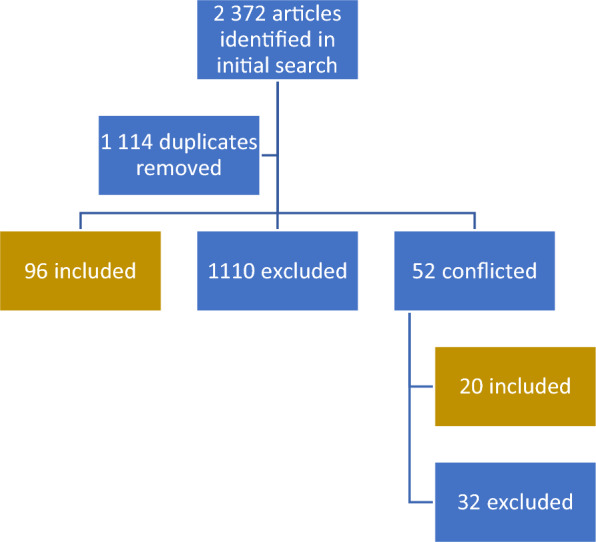
Process flow diagram

We structured the review’s findings in two main sections: quantitative studies (n = 60) and qualitative (n = 56) studies. We provide the study country, sample size, and research design in each section. We then provide a summary of findings and an overview of thematic areas appropriate for a scoping review. Please refer to Fig. 1 for an overview of the research process.

### Infertility in Africa: quantitative studies (n = 60)

#### Background variables summary

Sixty publications report on the psychosocial implications of infertility in developing countries through either quantitative or mixed methods. Of these, most were conducted in Nigeria (*n* = *27*), followed by Ghana (*n* = *10*), South Africa (*n* = *6*), Tunisia (*n* = *4*), Egypt (*n* = *3*), and Mali, Rwanda, and Sudan (*n* = *2*). Other countries in which single studies were conducted include Ethiopia, Malawi, Morocco, and Uganda.

Sample sizes varied considerably, with studies involving individual participants (*n* = *52*) having sample sizes ranging from 24 to 1 083 (*x̄* = *272.17, σ* = *241.33*). Studies with couples (*n* = *8*) had samples ranging from 50 to 600 (*x̄* = *264.13, σ* = *219.98*) couples. Of the individual participant studies, 48 studies assessed women, each with a mean of 256.94 participants (*σ* = *229.00*), while ten assessed men, each with a mean of 145.10 participants (*σ* = *144.53*). There were 17,753 participants in total, of which 12,076 were women, 1451 were men, and 2113 were couples.

In studies with individual women, 9401 presented with either primary or secondary infertility (*x̄* = *191.86, σ* = *145.15*), and 3301 were either voluntarily childless (i.e., using contraception) or had no trouble conceiving (*x̄* = *194.18, σ* = *233.33*). In studies with individual men, 947 presented with either primary or secondary infertility (*x̄* = *94.70, σ* = *92.40*), and 504 were either voluntarily childless or had no trouble conceiving (*x̄* = *126.00, σ* = *43.37*). In studies involving couples, 2013 couples presented with either primary or secondary infertility (*x̄* = *251.63, σ* = *226.88*), and 100 were either voluntarily childless (i.e., using contraception) or had no trouble conceiving, appearing in a single study.

Studies often made use of more than one study design. As shown in Appendix 1, 37 studies used a cross-sectional study design, and 19 used a comparative study design. Twelve studies used questionnaires as a primary data collection method, and 5 used in-depth interviews (as part of mixed methods studies). In addition, four studies were descriptive in nature, three were RCTs, one was a case–control study, one was an overview of a developing intervention, and one was unspecified.

#### General psychosocial research

In this section, we report on general psychosocial research among patients with infertility in African countries. Research on infertility in Africa covers several psychosocial domains, such as quality of life (QOL), factors associated with ART uptake, psychological well-being, coping, beliefs and knowledge, and attitudes towards adoption, surrogacy, and ART.

Comparisons between fertile and infertile women in Nigeria and Egypt indicate that infertile women report significantly poorer QOL and higher symptoms of depression than fertile women [19–25]. Women with infertility in Uganda reported poor QOL [26], while in Tunisia and Sudan, women with infertility reported lower QOL than their male partners [27, 28]. Studies reported on the prevalence of symptoms of depression, with prevalence rates ranging from 20 to 62%, depending on the sample and criteria of assessment [29–39]. Men with infertility also report significantly more symptoms of depression and anxiety than their fertile counterparts [40]. Therefore, researchers investigated the correlates of depressive symptoms. These symptoms were positively correlated with age and duration of infertility [41]. Another study found that religion, education, and monthly income were associated with depression severity [38].

Findings regarding symptoms of anxiety were somewhat contradictory. Naab et al. [42] found low levels of anxiety among infertile women in Ghana. In contrast, [43] Obajimi et al. [43] reported that almost half of their sample of infertile women attending a fertility clinic in Nigeria reported anxiety. Ofovwe, Aziken and Madu [44] compared infertile and pregnant women in Nigeria and did not find a significant difference between these groups of women in terms of overall psychological dysfunction. However, they found a significant difference in psychological dysfunction when they compared women with primary infertility to those with secondary infertility, with those indicating primary infertility reporting greater psychological dysfunction than those with secondary infertility [44].

Researchers also reported on distress among patients with infertility. Distress was reported by 17% of couples seeking fertility treatment in Ghana [17] and 20% of women seeking treatment in Mali [45]. Naab, Brown, and Heidrich [42] reported high levels of infertility-related stress among infertile women in Ghana. They also found that fertility beliefs were associated with infertility-related stress (21%), depressive symptoms (11%), anxiety (8%), social isolation (6%), and perceived stigma (5%). In South Africa, males and females with infertility reported significantly more distress than their fertile counterparts [46, 47]. In Tunisia, women reported significantly higher psychological distress than men across domains, including symptoms of anxiety, depression and self-esteem [48]. Another study conducted in Tunisia found that women with primary infertility were significantly more stressed than those with secondary infertility [49]. Researchers in Ethiopia reported a prevalence of infertility-related stress of 93% and identified age, marital status, motherhood status and duration of infertility as significant correlates of infertility-related stress [50]. Donkor and Sandall [51] reported that women with higher levels of education reported less infertility-related stress.

Studies reported on broader psychosocial experiences associated with infertility. For example, Anokye et al., [17] reported that among couples receiving fertility treatment in Ghana, 40% reported that they felt their lives were on hold; 28% indicated that infertility resulted in low self-esteem; 56% stated that they believed that infertility resulted in social exclusion; 41% indicated that they were subjected to verbal and physical abuse and 3% indicated that infertility led to marriage breakdown. Studies also report on self-esteem among women with infertility. For example, women with infertility report significantly lower self-esteem than controls [21]. In Egypt, infertile women reported lower rates of self-esteem, sexual satisfaction, and sexual self-esteem than women with children [52].

Relationships among patients with infertility were also explored quantitatively. A study comparing relationships in couples with primary infertility, secondary infertility, and fertile couples found that primary infertile women were most likely to have separated from a steady sexual partner and that men in primary and secondary infertile relationships were more likely to report multiple sexual partners and lack of condom use than men in fertile relationships [53]. Thirty-one percent of women attending an infertility clinic in Nigeria reported that they experienced intimate partner violence due to infertility [54]. Studies demonstrate the impact of infertility on relationships. Infertile women in Mali reported feeling more social pressure for pregnancy than fertile women, primarily from their husbands and female friends [55]. Women in Ghana shared that they preferred keeping information about their infertility to themselves and not disclosing it to others [56]. Larsen and colleagues [57] found that women with problems with fertility in Nigeria were less likely to still be married and were treated differently by their husbands, mother-in-law and the community. Orji et al. [58] surveyed 236 women with secondary infertility and found that 38.9% had divorced and remarried because of infertility. Of these, 78% reported that their husbands’ families abused them, 54% reported that their husbands took another wife, and 39% reported that they were accused of being a witch. Two more studies found that women with infertility experienced a deficit in supporting relationships with their spouse [23, 25] and spouse’s family and that experiencing discrimination from the community predicted psychiatric morbidity [23]. In Ghana, Nyarko and Amu [59] found that 72% of infertile women respondents reported difficulties in the stability of their marriages, resulting in disagreements. A study conducted in Sudan found that support impacted the spousal relationship, social pressure and coping with faith and non-faith-based practices [27].

Stigma was explored in several studies in Ghana. Naab, Brown & Heidrich [42] reported low levels of perceived stigma and social isolation among infertile women in Ghana. Another study conducted in Ghana found that stigmatisation was negatively correlated with fertility QOL and positively correlated with active-avoidance coping [60]. A survey among 615 women receiving infertility treatment in Southern Ghana reported that 64% of the sample reported feeling stigmatised and that higher levels of perceived stigma were associated with increased infertility-related stress [51].

Attitudes towards infertility, ART, alternate medicine, motivation for parenthood, fertility beliefs and consequences of infertility were also explored in African research. Akande, Dipeolu, and Ajuwon [61] found that 52% of their sample of patients receiving fertility treatment at a clinic in Nigeria held negative views of ART, despite using it. A study among 166 women diagnosed with infertility in Nigeria reported that 137 of these participants stated that they would embrace ART if it were offered to them, but 29 stated they would not, citing religion, fear of side effects, failure and high costs for this decision [62]. Another study conducted in Ilorin, Nigeria, found high levels of awareness of ART among infertile couples seeking fertility treatment but also found that most were unwilling to use surrogacy [63]. Among women seeking fertility treatment in Ibadan, Nigeria, 58.3% reported being aware of IVF, and 35.2% reported being aware of surrogacy [64]. Similarly, research on artificial donor insemination in Nigeria indicates low levels of awareness and acceptability among infertile males and females [65].

In addition, the majority of women sampled at fertility clinics in Lagos, Nigeria (85.7%) indicated that they knew of adoption. However, only a third (33.7%) reported that they were willing to consider adoption [66]. Women with infertility in Nigeria identified family constraints and culture as reasons they would not adopt [67]. Similarly, a sociological analysis of 400 women with infertility in Nigeria showed a strong belief among participants that spirituality played an important role in resolving infertility [68]. One study explored motives for parenthood among males and females seeking fertility treatment in South Africa [69]. Studies also explored knowledge about infertility, indicating poor levels of knowledge [70–72]. A survey of 600 couples receiving fertility treatment in Nigeria found that infertility was perceived as being attributed to destiny / supernatural powers (17.1%), a woman’s problem (15.6%) or a threat to males’ lineage (14.3%) [72].

Barriers to ART were identified in the research. The cost of ART was identified as a barrier to treatment [61]. A study among infertile Sudanese women found that 43.3% reported using alternate self-management methods to conceive, such as herbs and religious prayer. Most reported that the cost of ART was a barrier to ART [73]. Access to health care, including transportation issues, was identified as a barrier to infertility treatment among women in South Africa [71].

Studies described few counselling or psychological interventions to support women with infertility in Africa. However, two articles described such interventions. Aiyenigba et al. [74] described using the Fertility Life Counselling Aid in Nigeria, which uses Cognitive Behaviour Therapy to manage psychological morbidity associated with infertility. Naab et al. [75] tested the feasibility of a culturally adapted depression intervention among women with infertility in Ghana. Researchers found an improvement in women’s psychosocial health after the intervention of their programme, Oh Happy Day Classes (OHDCs).

Studies explored the role of non-psychologically trained staff in counselling and supporting patients seeking ART. In Morocco, Zaidouni et al. [76] conducted a comparative study and concluded that nurses effectively supported patients seeking ART treatment. A randomised controlled trial conducted in South Africa showed that patients who received psychological support delivered by embryologists were not better equipped to cope with fertility treatment than those who received no formal support at all [77]. However, those who received counselling reported significantly fewer symptoms of anxiety and higher use of problem-focused coping strategies than the control group [77].

We searched for publications describing the development of measures and exploring psychometric properties of measures among people seeking fertility treatment. We found one study that described the development of the Social Pressure for Pregnancy Scale and examined its psychometric properties among women in Mali, West Africa [55]. Findings reveal that the scale has good psychometric properties and can be used in future infertility studies, especially in relation to depression [55].

Studies also explored coping with infertility among patients in Ghana [56, 78, 79], Nigeria [80] and Mali [45]. In Nigeria, a lack of support was a significant predictor of symptoms of depression and anxiety for women with infertility [81]. Some women reported that their husbands played an important role in helping them cope with infertility [56] and were more likely to consider adoption if their spouse supported adoption [66]. In Ghana, women with infertility used their religion as a means of coping [56]. In Nigeria, women said they first sought help from a traditional or faith-based healer [82]. In a study conducted in Nigeria, all[83] participants reported that they sought spiritual solutions to their infertility. Spirituality was often seen as a solution to infertility, and traditional treatment methods are preferred to ART [84]. Fatoye et al. [85] found that Nigerian men’s spirituality was linked with lower anxiety symptoms.

The review of these studies showed that few explore the needs of infertility patients in Africa. Two studies in Nigeria highlight patients’ strong desire to carry their own biological child [82, 83]. In a study conducted in Sudan, participants reported that the lack of biological offspring left them with a feeling of ‘something missing’ [27]. We did not find studies that reported on the broader needs of patients. However, Awoyinka and Ohaeri [86] reported that 18% of their sample reported feeling that there was a lack of support from nurses when treatment failed, and 25% indicated that friends were unsympathetic and offered unhelpful suggestions.

### Qualitative articles about infertility in Africa

Fifty-six qualitative studies were identified in this review. The reviewed studies represented diverse populations, including thirteen studies in Ghana, eleven in Nigeria, six in Malawi, four in The Gambia, three each in South Africa, Cameroon, and Zimbabwe, two each in Mozambique, Senegal, Egypt and one each in Botswana, Kenya, Morocco, Rwanda, Sudan, Tanzania and Zambia. The majority (fifty-four) of the studies were exclusively qualitative in nature, with only three mixed methods studies included. The main source of data collection was semi-structured interviews, with some studies using key informant interviews, focus groups, document reviews, informal conversations, single case studies and participant observations to gain a holistic understanding of the issues related to infertility. Twenty-six studies addressed infertility from the perspective of women, six from men, fifteen from both men and women (including couples), seven from varied participants, one from clinicians and one from women and herbalists.

Regarding themes covered in the literature, twenty-two studies examined the broader perceptions of infertility, nine on male infertility, eight on community perceptions of infertility, four on infertility and ART, three on ART experience, three on infertility and health-seeking behaviour, one on marital relationships, one on polygamy, one on social support, one on holistic management of infertility, one on fertility education, one on religious perceptions of infertility, and one on perceived barriers to adoption as a response to infertility.

Studies showed that individual needs and sociocultural expectations shaped the desire to have children. In Africa, children symbolise advancement in one’s life course [87–96], the consecration of marital relations, continuity of family lineage, security in old age, labour, fulfilment of religious obligations, inheritance and social status [87, 90, 91, 93, 95, 97–104], companionship [90] and a connection between the living and the dead [105]. Furthermore, local interpretations of infertility went beyond the inability to have children to failure to have a male child or the socially expected minimum number of children [87, 89, 102, 106–108]. In addition, findings demonstrated adverse psychosocial implications on individuals, their marriages, and familial and social relations.

Sixteen studies reported on the psychological effects of infertility on women who reported feelings of sadness, stress, anxiety, loneliness, frustration, and depression from their inability to conceive [71, 89, 90, 97–99, 106, 109–118] and suicidal ideations [90, 114, 116]. In The Gambia, some women said infertility was their greatest grief [99]. In a Nigerian study, women reported depression to the point of being suicidal [114]. In addition, studies in Malawi, South Africa, Egypt, and Zimbabwe revealed the emotional impact of male infertility, whereby men declared feeling sadness, discomfort, anger, pain, depression, frustration, embarrassment, and a loss of identity [106, 119–122].

Societal values and norms contributed most significantly to the psychological turmoil experienced by infertile persons in Africa. Seventeen studies revealed that women bore the burden of a couple’s infertility on account of cultural beliefs and patriarchal and pronatalist societal norms [87, 95, 99, 102–105, 112, 117, 119, 122–128]. This gendered experience prompted women to seek treatment options more likely when compared to men [95, 111, 124]. Furthermore, studies in The Gambia and Zambia cited avoidance by men in seeking an infertility diagnosis from healthcare practitioners [105, 124].

With regards to marital relations, twenty studies reported on women’s experiences of ridicule, shame, and stigma; living in fear of isolation, reduced libido, financial strain, marital instability, polygamy, increased HIV risk, intimate partner violence, and divorce [87, 89, 90, 92, 98, 99, 102, 105, 107, 109, 113–116, 119, 122, 124, 126, 129, 130]. Childless Gambian women in polygamous marriages reported feeling less love, attention and financial support from their husbands than co-wives with children, igniting sadness, jealousy and poor self-image [98]. The same study found that the infertile women felt less pressure to conceive, citing better chances of conception whilst in the company of other pregnant women [98]. Interestingly, Dyer et al. [119], in their study of male infertility in South Africa, reported that men did not express concern over losing their relationships. At the same time, other studies on male infertility in Zimbabwe, Egypt, and Nigeria cited relational issues between the men and their wives [120] and poor sexual performance [121, 129].

Twelve studies reported on familial pressures on wives to conceive by their husband’s relatives [89, 90, 98, 99, 105, 109, 111, 115–117, 131, 132]. Nine studies revealed that women were mocked by their in-laws, relatives and community for their failure to conceive, referring to them as witches [98, 99, 101–103], useless [89, 106], empty basket, or barren sister [102, 114, 133]. In the Gambia, Malawi and Nigeria, four studies highlighted the unrealistic social pressures couples experience to have children as early as one year of marriage [95, 107, 111, 114]. While infertile men did not experience the same level of social stigma, studies reported feelings of loss of respect and ridicule of one’s manhood [87, 105, 106, 119, 134], family pressure to reproduce [119, 126], to take on a second wife [90, 98], or for a male relative to impregnate the wife [87, 135]. Two studies in Nigeria reported men’s concern over their wives taking on the blame by concealing their infertility diagnosis to prevent emasculating them [114, 136]. In contrast, a study from Ghana noted that men disclosed their infertility status to their families to relieve their wives of the pressure [117].

Sociocultural and religious beliefs influenced interpretations and misconceptions concerning infertility, attributing the condition to mystical, supernatural and natural factors [94, 98, 99, 103, 106, 112, 114, 119, 123, 124, 129, 134, 135, 137]. Six studies reported spiritual interpretations of infertility that included punishment from God, witchcraft, and displeased ancestors [91, 94, 103, 109, 119, 135]. Similarly, in Ghana, infertility was an adulterer’s curse from the ancestors [101, 102, 104]. Infertile individuals also experienced accusations of abortions, and overconsumption of contraceptives [90, 93, 99, 101–103, 106, 137], contraction of sexually transmitted infections [93, 99, 103, 114], multiple sexual partners [138]; and masturbation [102]. However, in Tabong and Adongo’s [102] study on the social meaning of infertility, urban participants identified likely natural causes, while rural participants described social causal factors.

These socio-cultural perceptions had a detrimental impact on the social status and identity of infertile individuals and couples in their communities. For example, in Ghana, four studies revealed that infertile couples were prohibited from assuming leadership positions and were not socially recognised [90, 101, 104, 139]. Similarly, in Mozambique, infertile women were barred from participating in cultural rituals associated with fertility, such as assisting in childbirth [112]. In Zambia, burial rituals for childless persons were performed differently from the norm and cited as shameful [105]. Comparative studies in Nigeria, The Gambia, Malawi, South Africa showed that women who attained motherhood achieved higher social status than their childless counterparts, who were considered socially inferior [71, 87, 89, 99, 123, 140]. Contrastingly, when exploring the experiences of infertility among urban women in Nigeria, Dierickx et al. [99] found that women with higher socioeconomic status appeared to exhibit greater agency over their marriages and social status compared to women of a lower status. These findings show how infertility devastates the psychosocial well-being of those who experience it.

There were limited studies on formal psychosocial counselling and interventions for infertile persons in Africa. Two studies discussed proper support, with one in Malawi reporting participants’ appreciation for compassionate clinical counselling received [134], while the other study in South Africa reported that persons seeking clinical treatment for fertility felt frustrated at the lack of compassion from the clinical team, critical to meeting their psychological and emotional needs [141]. Four studies reported participants’ criticism of the limited information available on infertility [105, 114, 119, 121, 124], while lack of good reproductive health services was noted in Mozambique [112]. Dierickx et al. [124] noted a local NGO and stakeholders’ engagement efforts in the Gambia to bring services closer to people experiencing infertility. Still, they highlighted husbands’ reluctance to attend and limited resources in rural communities.

The majority of studies found that individuals drew upon multiple informal sources of support to cope with the psychosocial implications of infertility based on their beliefs and preferences. Twelve studies revealed that participants gained support from family, friends, neighbours, peers, and colleagues [98, 99, 105, 115–117, 119, 124, 129, 130, 132, 142]. In Mozambique, Faria [132] found varying degrees of support, from emotional, financial, and instrumental to informational, spiritual, and treatment peer groups. Contention did exist among participants between disclosing their status to gain support at the risk of judgement and gossip [124, 132]. Individuals also sought help through biomedical support [87, 94, 102, 114, 124, 125, 129, 130, 143], spiritual/religious communities [87, 91, 94, 103, 105, 115, 116, 125, 129, 130, 132, 142], and traditional healers [87, 94, 102, 105, 107, 112, 119, 120, 125, 130, 133–135, 142, 144]. Mariano’s study [112] in Mozambique found that infertile women hardly attended hospitals and preferred local healers for treatment. In Nigeria, faith-based healers were considered the cheapest form of treatment, while traditional healers were as expensive as biomedical treatment [114]. Interestingly, seventeen studies reported on individual coping strategies that included avoidance [119, 142], keeping busy [91, 116, 130], trivialising husbands’ infidelity [97, 99], abstinence [129], transferring reproductive duty to the wife’s younger sister (female infertility) or male community member (male infertility) [87], fosterage [89, 91, 98, 103, 105, 130, 142, 145], societal conformity [103], economic advancements [89, 98, 99, 116], engaging with multiple sex partners [87, 97, 104, 105, 129, 145], and migration [89, 91, 117].

Fourteen studies called for better access to quality professional care and counselling [90, 112, 113, 116, 118, 129, 136, 141, 143, 146, 147]; in particular, emotional and psychological support for women [99] and training for providers to standardise treatment and counselling support [111, 124]. Beyond this, studies recommended that providers offer empathic care, given patients’ vulnerability and referrals to mental wellness services [141, 146]. On a macro level, there is a need for national policies to prioritise infertility as a serious public health issue [126], promote public awareness of infertility to eradicate myths, reduce stigma and boost reproductive healthcare attendance [27, 109, 111, 137, 139, 145, 146], and increase the worth of the girl child in society [96]. Dierickx et al. [111] posit the donor dependency on infertility treatment and its neglect by national governments and international funders who prioritise family planning as a major challenge. Hence, a multi-sectoral and holistic approach encompassing social, spiritual, economic, and political engagement may be required to address the psychosocial betterment of persons with infertility sufficiently.

## Discussion

The purpose of this scoping review was to map the psychosocial research on infertility conducted in Africa [148]. We found 116 articles that met our inclusion criteria. Nigeria produced the most studies (*n* = *38*), followed by Ghana (*n* = *23*) and South Africa (*n* = *9*). In addition, Nigeria produced the most quantitative studies (*n* = *27*), whereas Ghana produced the most qualitative studies (*n* = *13*).

The articles cover a broad range of thematic areas such as QOL; mental health; psychological experiences; self-esteem; sexual well-being; relationships; stigma, attitudes, beliefs, knowledge and perceptions relating to aspects such as ART, adoption and surrogacy; barriers to ART; needs and coping; health-seeking behaviour; infertility management and education; healthcare and accessibility to ART; psychological interventions, psychometry, and sociocultural and religious aspects of infertility. Qualitative and quantitative studies explored well-being and quality of life. These studies document factors such as the prevalence of symptoms of depression and anxiety and describe aspects of well-being among patients seeking infertility treatment. Quantitative studies also compared factors such as QOL, distress and relationships between men and women, fertile and infertile patients, and primary and secondary infertility. The psychological sequelae of infertility appear to be well-documented among women. However, this research among men is sparse.

There is rich literature on socio-cultural aspects of infertility, which is seen in studies that report how women are treated by their families, in-laws, and broader communities when they fail to conceive. However, there is a need for studies to be conducted in more diverse cultural settings. Similarly, there is some qualitative research on coping and support in the context of infertility but limited quantitative research. Further research exploring the coping, support and needs of patients is required.

We identified a gap in the literature regarding the design and assessment of psychological interventions for patients with infertility. Similarly, although there are several quantitative studies, there is a need to assess the psychometric properties of measures used in infertility studies and develop psychometric measures appropriate in these varying contexts.

Although every effort was made to locate studies relevant to this review, we concede that publications may have been overlooked. As we aimed to provide an overview of published literature in the field, we did not conduct any quality assessment of articles included in this review. However, articles in predatory journals or not peer-reviewed were excluded.

## Conclusion

In summary, over the past 22 years, 116 articles have been published on the psychosocial aspects of infertility in African countries. Most of these studies (81%) were conducted in Nigeria, Ghana, and South Africa. However, there is a need for more psychosocial research, particularly psychosocial interventions, on the African continent.

## Data Availability

Not applicable.
